# Patterns of Lipid Abnormalities in Obesity: A Comparative Analysis in Normoglycemic and Prediabetic Obese Individuals

**DOI:** 10.3390/jpm14090980

**Published:** 2024-09-15

**Authors:** Yazeed Alshuweishi, Abdulmalik A. Almufarrih, Arwa Abudawood, Dalal Alfayez, Abdullah Y. Alkhowaiter, Hamood AlSudais, Abdulaziz M. Almuqrin

**Affiliations:** 1 Department of Clinical Laboratory Sciences, College of Applied Medical Sciences, King Saud University, Riyadh 12372, Saudi Arabia; 2 Department of Family and Community Medicine, Prince Sultan Military Medical City, Riyadh 11159, Saudi Arabia; 3 King Salman Center for Kidney Diseases, Riyadh Second Health Cluster, Ministry of Health, Riyadh 14214, Saudi Arabia

**Keywords:** obesity, dyslipidemia, insulin resistance, prediabetes, normoglycemia

## Abstract

**Background:** Obesity is a growing global health concern, often accompanied by dyslipidemia, contributing to cardiovascular risk. Understanding the patterns of dyslipidemia in different glycemic states is crucial for targeted interventions. This study compares dyslipidemia patterns in normoglycemic and prediabetic obesity to improve clinical management strategies. **Methods:** The study analyzed the complete lipid profiles of 138 subjects, comparing the medians, prevalence, diagnostic performance, and risk assessment of each lipid parameter across 54 non-obese (NO), 44 normoglycemic obese (NG-OB), and 40 pre-diabetic obese (PreDM-OB) groups. **Results:** Elevated total cholesterol (TC) and low-density lipoprotein (LDL) were the most prevalent forms of dyslipidemia observed in obesity (45.35% and 43.53%, respectively). Stratification by glycemic status revealed that triglyceride (TG) levels were elevated in both the NG-OB and PreDM-OB groups, with a more marked increase in the latter group (73.07 mg/dL vs. 97.87 mg/dL vs. 121.8 mg/dL, respectively). Elevated LDL showed better diagnostic performance and higher odds ratios (OR) in the NG-OB group (AUC = 0.660, *p* = 0.006; OR = 2.78, *p* = 0.022). Conversely, low high-density lipoprotein (HDL) was more common and exhibited significant diagnostic performance, with higher OR values in the PreDM-OB group (AUC = 0.687, *p* = 0.002; OR = 3.69, *p* = 0.018). Importantly, all lipid ratios were elevated in obesity, with TC/HDL showing the highest predictive ability for prediabetes (AUC = 0.7491, *p* < 0.001). **Conclusions:** These findings revealed unique and common lipid abnormalities in normoglycemic and prediabetic obesity. Future research should explore the effects of targeted lipid management on obesity-associated complications.

## 1. Introduction

Dyslipidemia, defined by an abnormal distribution of lipids within the bloodstream, constitutes a major risk factor for cardiovascular diseases (CVDs) [1]. Despite the significant regional and population-based variability in the prevalence of dyslipidemia, the World Health Organization (WHO) recognizes it as an escalating concern in both developed and developing nations [2,3,4]. This is mainly due to rapid urbanization and lifestyle changes, including increased consumption of processed foods and decreased physical activity [5]. The prevalence of dyslipidemia exhibits substantial regional variation, with reported rates of 53% in the United States, 23.5% in the United Kingdom, 81.0% in Iran, and 36.51% in China [2,6,7,8]. In 2021, the prevalence of dyslipidemia in Saudi Arabia varied, with reported rates ranging from 12.5 to 43% based on reports from community-based studies [9,10], whereas hospital-based studies have indicated a considerably higher prevalence of 62.0% [5]. The high prevalence of dyslipidemia is often associated with other comorbid conditions such as diabetes, hypertension, and non-alcoholic fatty liver disease (NAFLD), which together exacerbate the risk of severe cardiovascular events [1,11,12]. Despite the known risks, many individuals with dyslipidemia remain undiagnosed or inadequately managed due to various barriers, including limited access to healthcare services, lack of awareness, and insufficient routine screening.

Dyslipidemia can be classified as primary, resulting from genetic disorders such as familial hypercholesterolemia, or secondary, which is multifactorial and associated with conditions like obesity, diabetes mellitus, hypothyroidism, and certain medications [13]. Furthermore, dyslipidemia manifests in various forms, which can occur individually or in combination [7,14,15]. These manifestations encompass elevated plasma levels of low-density lipoprotein (LDL) cholesterol, diminished levels of high-density lipoprotein (HDL) cholesterol, increased total cholesterol (TC), and elevated triglyceride (TG) levels [7,14,16]. Notably, each form of dyslipidemia contributes differently to cardiovascular risk. For instance, the impact of lowering plasma LDL levels on cardiovascular disease risk is especially evident in patients with familial hypercholesterolemia [17]. While traditionally, TGs and HDL have been debated as direct risk factors for CVDs, some studies highlight the complexity of their roles in cardiovascular health. It was shown that hypertriglyceridemia loses its significant association when adjusting for other risk factors, including diabetes, body mass index, glucose levels, hypertension, and smoking [18]. Similarly, others reported that high or normal levels of HDL were not protective against CVD events [19]. Thus, the distinctive roles of these lipid abnormalities necessitate a comprehensive understanding of their interplay and impact on cardiovascular health, emphasizing the need for tailored treatment approaches that address specific lipid profiles to effectively mitigate cardiovascular risk.

Obesity is a well-recognized major risk factor for dyslipidemia, and approximately 60–70% of obese individuals exhibit dyslipidemia, whereas 50–60% of overweight individuals are affected [20,21]. The typical lipid abnormalities associated with obesity include elevated levels of TGs, very low-density lipoprotein (VLDL), and apolipoprotein B (Apo B), while HDL and apolipoprotein A-I (Apo A-I) levels are generally low, and LDL levels are usually normal or slightly elevated [22,23,24]. Research shows that individuals classified as obese (≥30 BMI) experience a notably higher incidence of diabetes compared to those who are merely overweight (25.0–29.9 BMI) [25]. This distinction is essential, as it highlights the severity of obesity as a risk factor for metabolic disorders. Furthermore, the relationship between HbA1c levels and the risk of developing T2D is particularly alarming in adolescents with moderate to severe obesity [26]. In this demographic, the risk escalates exponentially, underscoring the urgent need for early intervention and preventive measures. These findings emphasize the importance of addressing obesity not only as a standalone issue but also as a significant contributor to the rising prevalence of diabetes, especially among younger populations.

In recent decades, Saudi Arabia has experienced profound lifestyle transformations characterized by an increase in sedentary behavior [27]. This transition has been accompanied by a corresponding rise in the prevalence of obesity and its associated comorbidities. Among the adult population, the obesity rate has surged to 24.9%, with an additional 30.5% classified as overweight [28]. The effects of these lifestyle changes extend beyond the realm of obesity, as demonstrated by the WHO ranking Saudi Arabia as the seventh-highest globally and the second-highest in the Middle East for diabetes prevalence [29]. An estimated 3 million Saudis have prediabetes, while 7 million individuals, accounting for 24% of the population, have been diagnosed with diabetes [30]. Insulin resistance, a key predictor of prediabetes, is commonly associated with dyslipidemia, and insulin-resistant patients both with and without type 2 diabetes (T2D) display qualitatively similar lipid abnormalities [31,32]. Notably, these dyslipidemic patterns are evident not only in adults but also in pediatric populations, suggesting that the adverse effects of insulin resistance on lipid profiles commence early in life [33,34]. The Insulin Resistance Atherosclerosis Study revealed that non-diabetic adults who later progressed to diabetes already exhibited a proatherogenic pattern of lipid abnormalities at baseline, indicating that dyslipidemia frequently precedes the development of insulin resistance and is an early event in the pathogenesis of type 2 diabetes (T2D) [35].

While obesity-associated insulin resistance is well-documented as being linked with dysregulations in lipid metabolism, not all obese subjects are prediabetics or develop T2D [36,37]. These individuals, who are obese but do not exhibit glucose intolerance or diabetes, may still present with distinct lipid abnormalities that differ from those observed in obese individuals with insulin resistance or T2D. The failure to include this subgroup in studies limits the understanding of how dyslipidemia manifests across different obesity phenotypes and may lead to less effective management strategies tailored to their unique lipid patterns. Additionally, published studies did not address the impact of prediabetes on the patterns of dyslipidemia among obese patients, which is crucial for understanding the interaction between obesity, glucose intolerance, and dyslipidemia. Without accounting for prediabetes, research may overlook important variations in obesity-associated lipid abnormalities that could influence treatment and management strategies. These gaps highlight the need for more detailed research that incorporates comprehensive lipid assessments and considers the impact of prediabetes on obesity-associated dyslipidemia to enhance the accuracy and applicability of findings related to dyslipidemia in obese populations. Therefore, the aim of this study was to investigate the patterns of lipid abnormalities associated with obesity within the Saudi population. Also, the study sought to compare the prevalence and specific patterns of dyslipidemia between normoglycemic and prediabetic obese patients. By elucidating these patterns, the study seeks to provide insights that could guide tailored management approaches for dyslipidemia and reduce cardiovascular risk in obese patients with different glycemic profiles.

## 2. Methods

### 2.1. Ethical Approval

This retrospective study was approved by the Institutional Review Board at Prince Sultan Military Medical City (PSMMC), Riyadh, Saudi Arabia, under approval number E-2165, approved on 14 September 2023.

### 2.2. Study Design

This retrospective observational study was conducted using medical records from the Family Medicine Clinics at Prince Sultan Military Medical City (PSMMC) between January 2022 and December 2023. The study included individuals aged 18–70 who had at least one recorded visit during the specified period. To ensure the relevance of the study to the specific research objectives, individuals with known diagnoses of type 2 diabetes (T2D), cardiovascular diseases, or who were pregnant were excluded. Eligible patients were classified based on body mass index (BMI) into two primary groups: non-obese (NO) group with a BMI < 30, and the obese (OB) group with a BMI ≥ 30 [38]. The OB subjects were further stratified based on their HbA1c levels into two groups: normoglycemic obese (NG-OB) (HbA1c < 5.7%) and pre-diabetic obese (PreDM-OB) (5.7% ≤ HbA1c < 6.5%) [39]. The studied subjects were classified as dyslipidemic if they exhibited any of the following lipid profile abnormalities: LDL of ≥130 mg/dL, TG levels of ≥150 mg/dL, TC of ≥200 mg/dL, HDL of <40 mg/dL, TC/HDL ratio of ≥6, TG/HDL ratio of >2, or LDL/HDL ratio of >2.5 [40].

### 2.3. Data Collection

Data were extracted from electronic medical records, ensuring that all information was anonymized to maintain patient confidentiality. The following variables were collected for each subject:

- Demographic and clinical data: Age, sex, and BMI were recorded. Weight and height were measured using a weighing scale and a portable stadiometer (Marsden H226, Marsden Weighing Group, South Yorkshire, UK). Patients were instructed to wear lightweight clothing during weight measurement. BMI was calculated by dividing body weight (kg) by height (m^2^). Medical history and current medications were reviewed to ensure adherence to the inclusion criteria.
- Laboratory Data: Biochemical data were available for each participant. Blood samples were collected routinely according to protocol and transported to the central laboratory. Quality assurance and control of all laboratory equipment were carried out regularly. Fasting Blood Glucose (FBG) and lipid parameters were measured using a Cobas-8000 autoanalyzer (Roche Diagnostics, Rotkreuz, Switzerland). HbA1c levels were measured using a Cobas-513 autoanalyzer (Roche Diagnostics, Rotkreuz, Switzerland).

### 2.4. Statistical Analysis

Nonparametric tests were used for statistical analysis of the studied subjects’ data since they were not normally distributed. The Mann–Whitney U test was utilized to compare between two study groups, and one-way ANOVA with the Kruskal–Wallis test was used to compare between three study groups. The results of the data analysis were presented as medians ± interquartile range (IQR) for continuous data and percentages for categorical data. The discriminative ability of HbA1c for lipid profile abnormalities in the studied groups was assessed using receiver operating characteristic (ROC) curve analysis and determination of the area under the curve (AUC). MedCalc software v23.0.2 was utilized to conduct the risk assessment analysis and GraphPad Prism v9.2.0 (GraphPad Software, Inc., 10.0.01, San Diego, CA, USA) was used to perform the statistical analysis. Statistical significance was determined by *p*-values that were less than 0.05.

## 3. Results

### 3.1. Baseline Characteristics of the Studied Population

This study involved 138 subjects, 54 of them were non-obese (NO), 44 were normoglycemic obese (NG-OB), and 40 were pre-diabetic obese (PreDM-OB). Females represented the majority of subjects in all three studied groups (Table 1).

The laboratory and demographic baseline characteristics of the studied groups were analyzed and presented in Table 1. The median ages of the NO, NG, and PreDM groups were comparable, with a trend towards an increase in the PreDM group. However, this trend did not reach statistical significance, indicating that while there may be a slight age difference, it is not substantial enough to impact the outcomes of the study. Furthermore, the data revealed a statistically significant difference between the groups in terms of BMI, white blood cell (WBC) count, and red blood cell (RBC) count, while no significant differences were noted in the remaining variables. Moreover, Table 2 describes the medications used by the study subjects and their comorbidities. There is a higher prevalence rate of hypertension (15%) among the PreDM-OB subjects compared to the NO and NG-OB groups (5.56% and 6.82%). Similarly, the prevalence of smoking was higher in the NG-OB (15.91%) and PreDM-OB (15%) groups compared to the NO group (5.56%) (Table 2).

### 3.2. Normoglycemic Obese Exhibited Different Forms of Lipid Abnormalities

**Figure 1:**
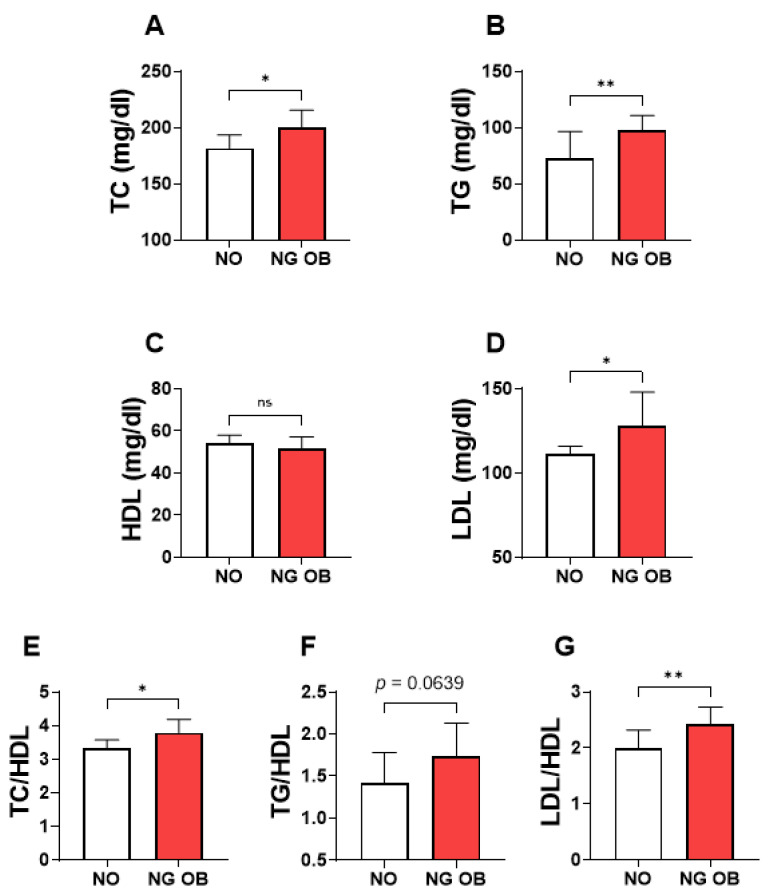
A comparative analysis of the lipid parameters between the non-obese (NO) group and normoglycemic obese (NG-OB) subjects. Changes in the levels of lipid markers between the NO and NG-OB groups are presented for TC (**A**), TGs (**B**), HDL (**C**), LDL (**D**), TC/HDL (**E**), TGs/HDL (**F**), and LDL/HDL (**G**). ns indicates not significant, while * (*p* < 0.05) and ** (*p* < 0.01) indicate significance.

Next, we examined the pattern of lipid abnormalities associated with normoglycemic obesity (NG-OB). Each lipid marker was compared between the NO patients and NG-OB patients. The analysis revealed a substantial increase in TC (NG-OB; 200.1 ± 170.0–228.2 vs. NO; 181.6 ± 154.7–201.2; Figure 1A), TGs (NG-OB; 97.87 ± 73.29–121.6 vs. NO; 73.07 ± 62.00–106.9; Figure 1B), LDL (NG-OB; 128.4 ± 106.1–153.6 vs. NO; 111.6 ± 93.97–127.7; Figure 1D), TC/HDL (NG-OB; 3.790 ± 3.358–4.700 vs. NO; 3.318 ± 2.898–3.821; Figure 1E), and LDL/HDL (NG-OB; 2.433 ± 2.067–3.171 vs. NO; 2.00 ± 1.723–2.503; Figure 1G) in the NG-OB group in comparison to the NO individuals. Although statistical significance was not achieved, the NG-OB subjects showed a substantial increase in the TG/HDL ratio compared to the NO counterparts (NG-OB; 1.733 ± 1.399–2.680 vs. NO; 1.422 ± 0.984–1.997; Figure 1F). No significant difference was observed in the HDL levels between these studied populations (Figure 1C).

### 3.3. TGs and HDL but Not LDL Were Significantly Altered in PreDM-OB Compared to the NO Group

**Figure 2:**
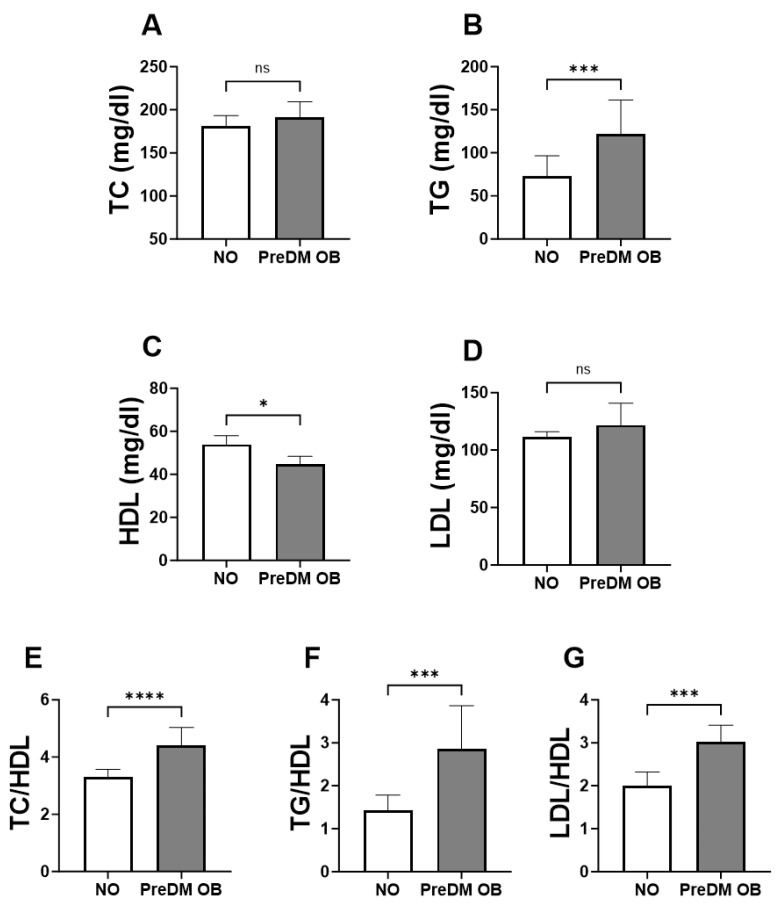
A comparative analysis of the lipid parameters between the non-obese (NO) and prediabetic obese (PreDM-OB) groups. Medians ± IQR of lipid markers in the NO and the PreDM-OB groups are illustrated for TC (**A**), TGs (**B**), HDL (C), LDL (**D**), TC/HDL (**E**), TGs/HDL (**F**), and LDL/HDL (**G**). ns indicates not significant while * (*p* < 0.05), *** (*p* < 0.001) and **** (*p* < 0.0001) indicate significance.

In the subsequent analysis, we investigated the combined effect of obesity and prediabetes on lipid parameters by comparing the lipid profiles of the non-obese group (NO) with obese subjects with prediabetes (PreDM-OB group). The results showed a significant reduction in HDL levels in the PreDM group compared to their NO counterparts (PreDM-OB; 44.66 ± 38.09–55.59 vs. NO; 53.94 ± 45.53–65.16; Figure 2C). In addition, the analysis showed a substantial increase in TGs (PreDM-OB; 121.8 ± 87.24–195.5 vs. NO; 73.07 ± 62.00–106.9; Figure 2B), TC/HDL (PreDM-OB; 4.416 ± 3.598–5.753 vs. NO; 3.318 ± 2.898–3.821; Figure 2E), TGs/HDL (PreDM-OB; 2.851 ± 1.671–4.738 vs. NO; 1.422 ± 0.984–1.997; Figure 2F), and LDL/HDL (PreDM-OB; 3.014 ± 2.070–3.660 vs. NO; 2.00 ± 1.723–2.503; Figure 2G) in the PreDM-OB subjects compared to their NO counterparts. No significant difference in TC and LDL levels was observed between these studied groups (Figure 2A,D).

### 3.4. TG and Lipid Ratios Were Significantly Higher in the PreDM Obese Compared to the NG Obese Group

**Figure 3:**
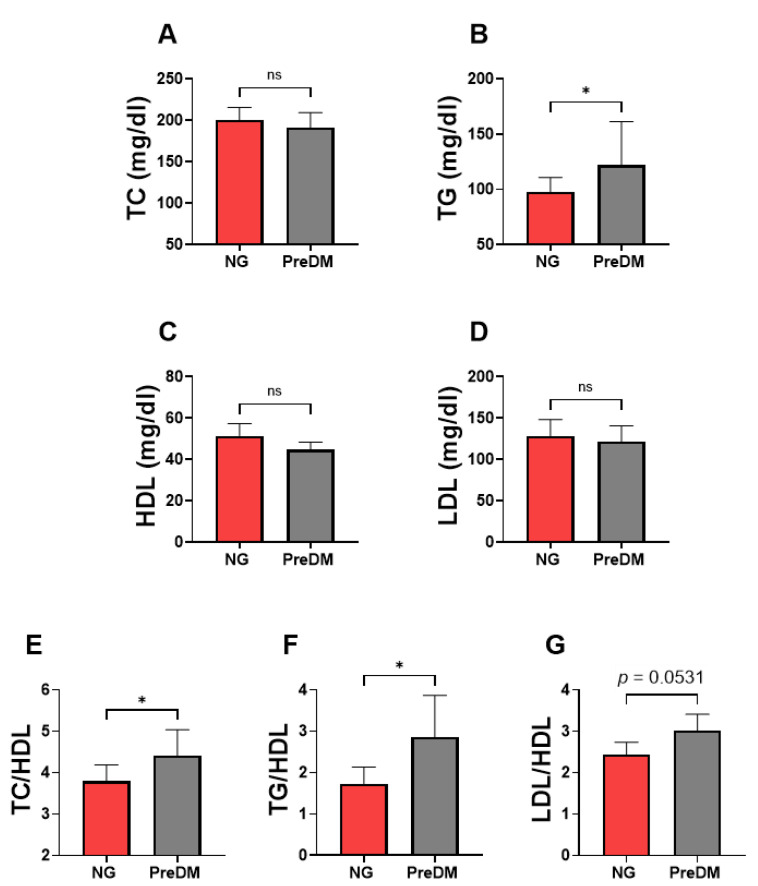
Assessing the lipid abnormalities between the normoglycemic obese (NG-OB) and prediabetic obese (PreDM-OB) groups. The changes in the levels of lipid markers between the NO and NG-OB groups are demonstrated for TC (**A**), TGs (**B**), HDL (**C**), LDL (**D**), TC/HDL (**E**), TGs/HDL (**F**), and LDL/HDL (**G**). ns indicates not significant while * (*p* < 0.05) indicates significance.

Finally, the current study compared the lipid abnormalities between the normoglycemic obesity and prediabetic obesity groups. Our observation showed a significant increase in levels of TGs (PreDM-OB; 121.8 ± 87.24–195.5 vs. NG-OB; 97.87 ± 73.29–121.6; Figure 3B), TC/HDL (PreDM-OB; 4.416 ± 3.598–5.753 vs. NG-OB; 3.790 ± 3.358–4.700; Figure 3E), and TGs/HDL (PreDM-OB; 2.851 ± 1.671–4.738 vs. NG-OB; 1.733 ± 1.399–2.680; Figure 3F) in PreDM-OB subjects compared to the NG-OB group. No significant difference in TC, HDL, and LDL levels was observed between these groups. It is worth noting that the LDL/HDL marker was substantially higher in the PreDM-OB group (3.014 ± 2.070–3.660) compared to their NG-OB counterparts (2.433 ± 2.067–3.171) with a *p*-value of 0.054, which is very near statistical significance (Figure 3G).

### 3.5. Differential Predictive Performance of Lipid Parameters for Different Obesity Phenotypes

**Figure 4:**
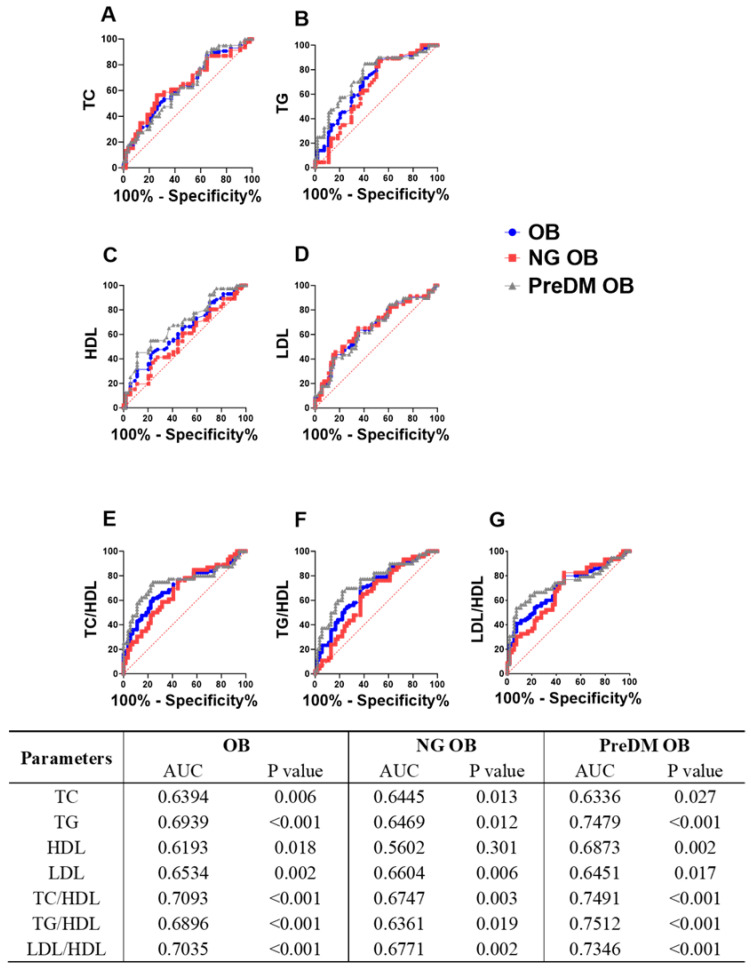
ROC curve analysis of lipid parameters for different obesity phenotypes. ROC curves of lipid parameters in obese (OB), normoglycemic obese (NG-OB), and prediabetic obese (PreDM-OB) groups are illustrated for TC (**A**), TGs (**B**), HDL (**C**), LDL (**D**), TC/HDL (**E**), TGs/HDL (**F**), and LDL/HDL (**G**). Area under the ROC curve (AUC) and *p* value of each lipid parameter is shown in table.

The discriminative capability of lipid profile parameters across all studied groups, including obese (OB), normoglycemic obese (NG-OB), and prediabetic obese (PreDM- OB) patients, was analyzed utilizing receiver operating characteristic (ROC) curve analysis (Figure 4). The results revealed that all lipid parameters exhibited a significant predictive ability to differentiate the OB group compared to the NO group, with TC/HDL showing the highest area under the curve (AUC) value, as shown in Figure 4E (AUC = 0.7491, *p* < 0.001). When we compared the predictive ability of lipid parameters between the NG-OB group and the PreDM-OB group, we observed the differential predictive ability of certain forms of dyslipidemia between PreDM-OB patients and NG-OB patients. Notably, all lipid parameters were better at differentiating PreDM-OB patients from NG-OB patients, except LDL, which showed an AUC value with PreDM-OB similar to the NG-OB group (Figure 4D; PreDM-OB AUC = 0.645, *p* = 0.017 vs. NG-OB AUC = 0.660, *p* = 0.006). Conversely, HDL was able to significantly discern PreDM-OB patients but not NG-OB patients (Figure 4C; PreDM-OB AUC = 0.687, *p* = 0.002 vs. NG-OB AUC = 0.560, *p* = 0.301).

### 3.6. LDL Is the Most Prevalent Lipid Abnormality among Obese Patients

Next, we assessed the prevalence of variant forms of dyslipidemia within the studied population. As shown in Table 3, the prevalence of all forms of dyslipidemia was higher in the obese group (OB) compared to the non-obese subjects (NO). In addition, several forms of dyslipidemia, including elevated TGs, low HDL, and high TG/HDL and LDL/HDL ratios, were more common among PreDM-OB subjects compared to the NG-OB group. Interestingly, the NG-OB group had a higher prevalence rate of elevated TC, LDL, and TC/HDL ratio compared to the PreDM-OB group.

### 3.7. TC/HDL Carries a Greater Risk of Developing Prediabetes among Obese Patients

As revealed in Table 4, the odds of developing multiple forms of dyslipidemia were significantly higher among PreDM-OB subjects (TG; OR = 5.11, *p* = 0.003), (L-HDL; OR = 3.69, *p* = 0.018) (TC/HDL; OR = 13.46, *p* = 0.001), (TG/HDL; OR = 5.82, *p* = 0.001) and (LDL/HDL; OR = 12.63, *p* < 0.0001) compared to the NG-OB group (TG; OR = 1.21, *p* = 0.757), (L-HDL; OR = 1.97, *p* = 0.236), (TC/HDL; OR = 5.56, *p* = 0.037), (TG/HDL; OR = 1.89, *p* = 0.241), and (LDL/HDL; OR = 5.03, *p* = 0.008). On the other hand, the analysis revealed that the chance of developing elevated levels of TC and LDL was higher in the NG-OB group (TC; OR = 3.33, *p* = 0.007 and LDL; OR = 2.78, *p* = 0.0223) compared to the PreDM-OB individuals (TC; OR = 2.22, *p* = 0.083 and LDL; OR = 2.32, *p* = 0.069).

## 4. Discussion

This study specifically investigates the effects of glycemic alterations on lipid metabolism within obese populations. Our approach focused on obesity rather than mere overweight, as obese subjects demonstrate a higher susceptibility to dysglycemia and an increased risk of developing T2D [25,26]. The present study sheds light on the metabolic complexities associated with obesity, highlighting the diverse patterns of dyslipidemia across different obesity phenotypes. Stratification of the obese population by glycemic status uncovered both shared and distinct dyslipidemic profiles (Table 5). This stratification not only highlighted the common metabolic disruptions prevalent in obesity but also brought to light unique lipid abnormalities associated with varying degrees of glycemic control, underscoring the complex interplay between obesity and metabolic health. Notably, hypertriglyceridemia was observed in both normoglycemic and prediabetic obesity, with a more pronounced increase in the prediabetic group (Figure 2). However, distinctive differences were observed in other lipid profiles, with elevated LDL levels predominantly found in normoglycemic obese individuals (Figure 1), while lowered high-density lipoprotein (HDL) levels were only observed in prediabetic obesity (Figure 2). Notably, normoglycemic obesity exhibited multiple forms of lipid abnormalities, including elevated TGs and LDL as well as high lipid ratios (TC/HDL, TGs/HDL, and LDL/HDL) (Figure 1). Notably, the lipid ratios of TC/HDL, TGs/HDL, and LDL/HDL were consistently elevated in both normoglycemic and prediabetic obesity (Figure 1, Figure 2 and Figure 3). Monitoring these lipid ratios is essential in routine clinical assessments of obese individuals, as they offer valuable predictive insights beyond traditional lipid measurements. Overall, these findings indicate that obese individuals with normal glycemic status remain at substantial risk for dyslipidemia. This underscores the need for comprehensive lipid profiling in obese patients who exhibit normoglycemia. The presence of prediabetes in the context of obesity further aggravates lipid disturbances, emphasizing the critical role of incorporating glycemic status into dyslipidemia assessments. By integrating these factors, healthcare professionals can develop more tailored and effective management strategies to address the multifaceted nature of lipid abnormalities in obese patients. This approach ensures a more comprehensive understanding of dyslipidemia risk and facilitates better-targeted interventions, ultimately enhancing patient outcomes.

Normoglycemic obesity exhibited multiple forms of lipid abnormalities, including elevated triglycerides (TGs) and LDL, alongside high lipid ratios such as TC/HDL, TGs/HDL, and LDL/HDL. The median concentrations of TGs and LDL among obese patients were 103.2 mg/dL and 126.8 mg/dL, respectively; these levels were significantly elevated compared to the non-obese group, with increases of approximately 30% for TGs and 12% for LDL. It is important to note that although these values do not exceed the high thresholds defined by the reference range [41], they fall within the borderline range, indicating a potential risk for cardiovascular issues if not adequately managed. Consistent with our findings, dyslipidemia was observed in subjects with normal glucose tolerance (NGT) [42]. However, those dyslipidemic patients, even with normal glucose tolerance, already exhibited impaired glucose metabolism in the form of impaired β cell function [42]. This suggests that the presence of dyslipidemia precedes the development of prediabetes and type 2 diabetes (T2D). It was reported that individuals with hyperlipidemia are more than three times as likely to develop T2D compared to those with normal lipid levels [43]. The observed dyslipidemia in normoglycemic individuals suggests that lipid abnormalities could be an early indicator of metabolic dysfunction, warranting proactive monitoring and intervention to prevent the progression to more severe conditions such as prediabetes and T2D.

The current study demonstrated that individuals with prediabetic obesity exhibited higher TG levels and lower HDL levels compared to their non-obese counterparts. Moreover, the odds of having elevated TG levels and reduced HDL levels were significantly higher in prediabetic obese individuals than in those with normoglycemic obesity. These findings indicate that not all forms of dyslipidemia are directly associated with obesity-associated insulin resistance. Supporting these observations, multiple studies found that hypertriglyceridemia and low HDL levels were more prevalent in patients with type 2 diabetes (T2D) compared to the general population, while high low-density lipoprotein (LDL) levels did not differ significantly between these groups despite matched age, sex, and BMI [44,45,46,47]. This indicates that hypertriglyceridemia and low HDL are likely causal factors to the development of T2D. Early studies reported that low HDL cholesterol levels predict the development of T2DM in prediabetics [48,49]. Research from animal and human studies demonstrated that HDL plays a key role in regulating insulin secretion by preventing cellular lipid accumulation, alleviating endoplasmic reticulum (ER) stress, and preventing apoptosis in pancreatic beta cells [50,51,52]. These insights emphasize the need for targeted lipid management in prediabetic individuals, focusing on normalizing TG and HDL levels to potentially mitigate the progression to T2D. Further investigation is required to delineate the specific pathways through which dyslipidemia affects insulin resistance and diabetes risk, as this understanding may facilitate the formulation of more targeted and effective preventive and therapeutic interventions.

Moreover, both phenotypes of obesity, normoglycemic and prediabetic, demonstrated elevated lipid ratios, showing the most consistent alterations as they were higher in normoglycemic and prediabetic patients. Lipid ratios have been increasingly recognized for their clinical relevance, largely due to their predictive capabilities. Furthermore, in a large cohort of participants who were free from cardiovascular disease (CVD), the risk of developing atherosclerotic cardiovascular disease was notably elevated for individuals with a TC/HDL ratio of ≥4.2 [53]. The TC/HDL ratio is linked to cardiovascular risk, with higher ratios being associated with an increased likelihood of cardiovascular morbidity and mortality [54]. Furthermore, elevated TG/HDL-C ratios are strongly associated with insulin resistance and metabolic syndrome, serving as a reliable indicator of these conditions in both general and high-risk populations [55,56]. In a large longitudinal cohort study, a positive association was observed between the LDL/HDL ratio and the risk of developing prediabetes among the adult Chinese population [57]. In the current study, TC/HDL carried the greatest risk for prediabetes as revealed by calculated ORs and showed the best diagnostic performance in discriminating prediabetic obese patients among other lipid ratios. Previous studies in the context of obesity often overlooked and did not thoroughly examine these lipid ratios, despite their critical importance in predicting prediabetic and cardiovascular risks. The focus has typically been on traditional markers such as TC, TGs, HDL and LDL, while ratios such as TC/HDL provide a more nuanced understanding of lipid-related risk factors. Addressing this gap, the current study highlights the value of these lipid ratios as vital components of complete lipid profiles, emphasizing the need for their inclusion in future research and clinical evaluations.

The current study is not without limitations. Firstly, as a single-center study, it may restrict the generalizability of the findings to wider populations with varied demographics and healthcare environments. Secondly, the retrospective nature of this study limits the ability to establish causality between variables, making it challenging to determine the direction of the relationships observed. Additionally, there is a lack of data regarding obesity-related indices such as waist circumference and other body composition measurements, which are critical for a comprehensive assessment of obesity and its associated health risks. These indices provide valuable insights into visceral fat distribution and its impact on cardiovascular health. The lack of such indices may lead to an incomplete understanding of the relationship between obesity and lipid abnormalities. Furthermore, the study may have omitted potential confounding factors such as lifestyle behaviors, dietary patterns, and physical activity levels, which could influence both lipid profiles and cardiovascular risk. Future studies should aim to address these limitations by including multicenter designs, prospective data collection, and a broader range of obesity-related and lifestyle variables to enhance the robustness and applicability of the findings.

## 5. Conclusions

In summary, this study demonstrates distinct dyslipidemia patterns in normoglycemic versus prediabetic obese individuals, adding to the growing body of evidence that underscores the complexity of lipid profiles in obesity. Elevated TC and LDL were prevalent across obesity groups, with significant variations in TG and HDL levels based on glycemic status. Additionally, all lipid ratios were significantly elevated in obesity, with the TC/HDL ratio showing the highest predictive capacity for prediabetes. These findings reinforce the need for a comprehensive approach to risk assessment that includes evaluating glucose metabolism and detailed lipid profiles. Future research should focus on elucidating the mechanisms behind these associations and developing targeted interventions that address both glycemic and lipid abnormalities in obese individuals.

## Figures and Tables

**Table 1:** Baseline characteristics of the studied population.

Parameter	NO Group	NG-OB Group	PreDM-OB Group	*p*-Value
BMI (kg/m^2^)	26 (22–27)	36 (34–38)	35 (33.25–38)	<0.0001
Age (Years)	39.5 (27.5–46.5)	35 (28.25–42.75)	43.5 (34.5–51.75)	0.057
Gender (female), n (%)	38 (70.37%)	32 (72.73%)	26 (65%)	-
HbA1c (%)	5.37 (5.12–5.50)	5.47 (5.30–5.55)	6.02 (5.83–6.16)	<0.0001
WBC (×10^9^/L)	6 (4.92–6.82)	7.35 (5.52–8.32)	6.25 (5.15–7.6)	0.010
Monocyte count (×10^3^/µL)	0.47 (0.37–0.57)	0.55 (0.43–0.64)	0.44 (0.38–0.55)	0.056
Basophil count (×10^3^/µL)	0.04 (0.025–0.05)	0.045 (0.03–0.06)	0.04 (0.03–0.062)	0.287
Neutrophils (×10^3^/µL)	2.92 (2.08–3.70)	3.64 (2.52–4.50)	2.74 (2.21–4.13)	0.087
Eosinophils (×10^3^/µL)	0.135 (0.08–0.20)	0.16 (0.12–0.24)	0.17 (0.10–0.24)	0.286
TSH (mIU/L)	1.9 (1.50–2.78)	2.34 (1.84–3.03)	2.1 (1.36–3.76)	0.561
25-hydroxyvitamin D (ng/mL)	50.7 (39.95–67.6)	42 (31.7–55.8)	43.25 (27.65–71.1)	0.071
RBC count (×10^12^/µL)	4.71 (4.4–5.12)	4.90 (4.59–5.32)	4.59 (4.33–5.00)	0.046
Hb (g/dL)	13.2 (12.4–14.18)	13.35 (11.53–15.03)	12.90 (11.10–13.43)	0.164
ALT (U/L)	13 (10–18.75)	15 (11–27.25)	15 (12–20.75)	0.220

WBC, white blood cell count; TSH, thyroid-stimulating hormone; RBC, red blood cell count; Hb, Hemoglobin; ALT, alanine transaminase.

**Table 2:** Comparison of comorbidities and medications in use and between the studied groups.

	NO Group	NG-OB Group	PreDM-OB Group
**Medications**			
GLP-1 agonist, n (%)	0 (0%)	15 (34.09%)	13 (32.50%)
Metformin, n (%)	1 (1.85%)	2 (4.55%)	8 (20%)
Iron supplementation, n (%)	3 (5.56%)	11 (25%)	10 (25%)
**Comorbidities**			
Hypertension, n (%)	3 (5.56%)	3 (6.82%)	6 (15%)
PCOS, n (%)	1 (1.85%)	2 (4.55%)	1 (2.5%)
Smoking, n (%)	3 (5.56%)	7 (15.91%)	6 (15%)

GLP-1, Glucagon-like peptide 1; PCOS, polycystic ovary syndrome.

**Table 3:** Prevalence of dyslipidemia among the studied groups.

Parameter	NO (%)	OB (%)	NG-OB (%)	PreDM-OB (%)
TC				
<200 mg/dL	76.92	54.65	50.00	60.00
≥200 mg/dL	23.08	45.35	50.00	40.00
TG				
<150 mg/dL	88.46	74.42	86.36	60.00
≥150 mg/dL	11.54	25.58	13.64	40.00
HDL				
≥40 mg/dL	88.46	74.42	79.55	67.50
<40 mg/dL	11.54	25.58	20.45	32.50
LDL				
<130 mg/dL	76.92	56.47	54.55	58.97
≥130 mg/dL	23.08	43.53	45.45	41.03
TC/HDL				
<6	96.15	74.42	81.82	65.00
≥6	3.85	25.58	18.18	35.00
TG/HDL				
≤2	86.54	66.28	77.27	52.50
>2	13.46	33.72	22.73	47.50
LDL/HDL				
≤2.5	92.31	61.18	70.45	48.72
>2.5	7.69	38.82	29.55	51.28

**Table 4:** The risk assessment analysis of dyslipidemia among the obese populations.

Parameters	Overall		NG-OB		PreDM-OB	
	OR	*p* Value	OR	*p* Value	OR	*p* Value
TC	2.77	0.0098	3.33	0.0070	2.22	0.0832
TGs	2.64	0.0524	1.21	0.7570	5.11	0.0026
L-HDL	2.64	0.0524	1.97	0.2360	3.69	0.0175
LDL	2.57	0.0169	2.78	0.0223	2.32	0.0692
TC/HDL	8.59	0.0048	5.56	0.0366	13.46	0.0011
TGs/HDL	3.27	0.0110	1.89	0.2405	5.82	0.0006
LDL/HDL	7.62	0.0003	5.03	0.0088	12.63	<0.0001

**Table 5:** Summary of main findings.

Characteristics	NO		NG-OB	NO		PreDM-OB
TC		<			-	
TGs		<			<	
HDL		-			>	
LDL		<			-	
TC/HDL		<			<	
TGs/HDL		-			<	
LDL/HDL		<			<	

NO: non-obese, NG-OB: normoglycemic obese, PreDM-OB: prediabetic obese, <: significantly higher level, >: significantly lower level, -: no significance.

## Data Availability

Data are available from the corresponding author upon reasonable request, and with permission of Prince Sultan Military Medical City (PSMMC).
